# Performance evaluation of machine learning algorithms for estimating reference evapotranspiration based on NASA POWER weather data: a case study in Nigeria

**DOI:** 10.3389/frai.2026.1801981

**Published:** 2026-04-10

**Authors:** Oluwaseun Temitope Faloye, Grace Awotoye, Oluwadamilare Oluwasegun Eludire, Oluwatobi Solomon Olaleye, Ayoola Olamitomi Oluwadare, Oluwafemi E. Adeyeri, Laemthong Laokhongthavorn, Viroon Kamchoom

**Affiliations:** 1 Department of Water Resources Management and Agrometeorology, Federal University, Oye-Ekiti, Ekiti, Nigeria; 2 Federal College of Education Okene, Okene, Kogi, Nigeria; 3 Faculty of Engineering and Applied Science, Cranfied University, Bedford, United Kingdom; 4 Department of Agricultural and Bioresources Engineering, Faculty of Engineering and Technology, University of Calabar, Calabar, Nigeria; 5 Department of Physical and Chemical Sciences, Faculty of Basic and Applied Sciences, Elizade University, Ilara Mokin, Ondo, Nigeria; 6 ARC Centre of Excellence for the Weather of the 21st Century, Fenner School of Environment and Society, The Australian National University, Canberra, ACT, Australia; 7 Excellent Centre for Green and Sustainable Infrastructure, School of Engineering, King Mongkut’s Institute of Technology Ladkrabang, Bangkok, Thailand

**Keywords:** decision trees, NASA POWER, Nigeria, Penman–Monteith, reference evapotranspiration, support vector machine

## Abstract

The Penman–Monteith (PM) method is recognized as the globally accepted approach for estimating reference evapotranspiration (ETo). However, its use is constrained in areas with limited or unavailable data. Predicting ETo using multiple support vector machine (SVM) kernels and decision tree (DT) ensembles with NASA POWER data is innovative, as previous SVM-based ETo prediction studies have relied primarily on linear kernels. This study aims to evaluate the performance of different machine learning (ML) models, specifically SVM and DT and their ensembles, using NASA Power data as input. For this purpose, ML models were trained using average values of the monthly climatic data (maximum and minimum air temperatures, relative humidity, and wind speed) from NASA POWER. ETo was used as the output variable and was calculated from ground-observed data using the PM method. The developed ML models underwent training and validation to determine ETo in areas with different weather conditions in Nigeria: Kano—dry weather, Onne—wet weather, and Ibadan—moderate weather. Thirty and 70 % of the data were used during training and validation, respectively. The SVMs used in this study include linear SVM, quadratic SVM, cubic SVM, fine Gaussian (FG) SVM, medium Gaussian (MG) SVM, and coarse Gaussian SVM. The decision trees include fine, medium, and coarse trees, along with their ensembles: bagged and boosted trees. The model performance was evaluated using various error metrics. The FG SVM model exhibited the most accurate and precise estimation of ETo, with root mean square error (RMSE) values of 0.38 and 0.599 mm during the training and testing phases, respectively. Additionally, the coefficient of determination (r^2^) was good, with values of 0.87 and 0.72 during training and validation. The FG SVM outperformed all other models across all study locations, demonstrating its robustness in predicting ETo despite the contrasting weather conditions. Overall, this study revealed that the integration of data from NASA POWER with FG SVM accurately estimated reference evapotranspiration, which is important for effective water resource management in areas where ground climatic data is unavailable.

## 1 Introduction

Evapotranspiration (ET) is one of the important hydrological components that plays a crucial role in the hydrological cycle of the ecosystem (Oki and Kanae, 2006; Jasechko et al., 2013). Based on the energy budget, it has been reported that approximately 50% of the net radiation absorbed by the Earth is converted to ET, while approximately 60% of the ET is returned to the atmosphere in the form of precipitation (Seneviratne et al., 2010; Nooni et al., 2023). These cyclic phenomena make ET an important indicator of atmospheric demand, which has a direct impact on the availability of water on Earth for crops (Fisher et al., 2017).

The accurate estimation of evapotranspiration is very important for the proper management of water resources and watersheds, both from agricultural and hydrological perspectives. This is because ET is commonly used to estimate crop water requirements and aids in the scheduling of irrigation, including when to apply water, the amount of irrigation needed, and drought management (Raziei and Pereira, 2013). Evapotranspiration has also been used to identify regions prone to drought and is an important field of research related to climate change (Croitoru et al., 2013). Quantifying crop evapotranspiration must frequently be preceded by determining reference evapotranspiration (ETo), which has been defined by Doorenbos and Pruitt (1977) as the rate of evapotranspiration from an extensive area covered with grass that is 0.08 to 0.15 m tall, uniform, actively growing, completely shading the ground, and under adequate soil–water conditions. Allen et al. (1998) elaborated on the concept of ETo by referring to it as a hypothetical reference crop with an assumed height of 0.12 m, a fixed surface resistance of 70 s m^−1^, and an albedo of 0.23. Due to the broad areas where reference evapotranspiration is important (drought, irrigation, and climate-related studies), its computation remains imperative. Its estimation can be performed directly and accurately using field-installed equipment like lysimeters or indirectly computed using mathematical models that yield good outputs (Alves Sobrinho et al., 2011).

However, the cost of constructing and maintaining lysimeters is high, limiting their use by researchers (Xu and Chen, 2005; Melo and Fernandes, 2012). This cost limitation has led researchers to rely on empirical approaches for computing ETo. One of the empirical methods that has stood the test of time and is considered accurate and precise is the FAO Penman–Monteith method. This approach has been shown to produce accurate ETo values in many regions and climates worldwide (Allen et al., 2006). Moreover, when compared to other empirical methods, it has been globally reported and accepted to outperform alternative methods for estimating ETo (Cai et al., 2007). Despite the accurate estimation of ETo typically obtained from the Penman–Monteith approach, the availability of climatic data remains a limitation in some parts of the world, such as Nigeria, where access to climatic data is a challenge. Moreover, since a large amount of data is required, this method may become a limitation for poor farmers who also lack the financial capacity to purchase climatic data, even if available from some agencies, and may not be financially able to buy an automatic meteorological station (Borges et al., 2012). Due to the challenges of data limitations often encountered with this approach, some researchers (Kim et al., 2022; Adeboye et al., 2009; Bhavya et al., 2025) have recommended the use of limited climatic data to overcome the challenge of the large data requirement for the Penman–Monteith approach. In Nigeria, there are areas without weather stations, resulting in a lack of ground data for estimating reference evapotranspiration. This lack of climatic data may affect agricultural productivity, irrigation scheduling, and drought and flood predictions in such areas.

Recently, some researchers (Aboelkhair et al., 2019; Tayyeh and Mohammed, 2024) have suggested that when weather station data are unavailable, satellite weather data produced from global atmospheric models can serve as alternatives. When climatic data are unavailable or scarce, such data are commonly obtained from atmospheric models like The Climate Hazards Group InfraRed Precipitation with Station data (CHIRPS), Climate Forecast System Reanalysis (CFSR), National Center for Environmental Prediction/National Center for Atmospheric Research (NCEP/NCAR), and The National Aeronautics and Space Administration Prediction of Worldwide Energy Resource (NASA POWER) (Funk et al., 2015; Schneider et al., 2014; Kanamitsu et al., 2002; Faloye et al., 2025). Among these atmospheric models, NASA POWER was selected since it provides global coverage with a resolution of 0.5 degrees latitude by 0.5 degrees longitude (Duarte and Sentelhas, 2020; Monteiro et al., 2004; Bai et al., 2010). Moreover, its interface is user-friendly, and the climatic data is globally available and can be downloaded from any part of the world.

Despite the availability of climatic data from atmospheric models, studies exploring the possibility of estimating reference evapotranspiration using these models, particularly NASA POWER climatic data, are limited. Moreover, studies integrating ETo estimation with machine learning (ML) algorithms are even fewer, highlighting the innovation of this study. Additionally, the use of artificial intelligence in agriculture is increasingly gaining attention and has been applied to solve a wide range of agricultural problems (Naqi et al., 2025; Khan et al., 2020; Kolivand et al., 2019; Saba et al., 2017). Therefore, the support vector machine (SVM), decision trees (DT), and linear regression model approaches were adopted. The multiple linear regression (MLR), DT, and SVM methods were selected because they have all been reported to accurately predict reference evapotranspiration (Kim et al., 2022; Bhavya et al., 2025; Heramb et al., 2023). In addition, previous studies that applied SVM to estimate ETo used only the linear type. To date, there is a dearth of information on the performance of other types of SVM kernels like polynomial, cubic, fine Gaussian, medium Gaussian, and coarse Gaussian Similarly, various decision tree types (fine, medium, and coarse trees) and their ensembles (boosted and bagged trees) have not been tested for estimating ETo from climatic models obtained from atmospheric models. The comprehensive evaluation of these SVM kernels, DT types, and their ensembles is innovative and important for advancing knowledge and selecting the best ML algorithm for ETo estimation in areas with different weather conditions in Nigeria.

Therefore, this study aims to estimate ETo in regions with contrasting weather conditions in Nigeria using NASA POWER data and various ML methods. The key objectives of this study were to (i) develop machine learning-based ETo prediction models such as SVM, RF, and their ensembles (BT and BGT) for areas with contrasting weather conditions in Nigeria, and (ii) evaluate the spatio-temporal performance characteristics of the ML models for ETo predictions.

## 2 Materials and methods

### 2.1 Study area

**Figure 1:**
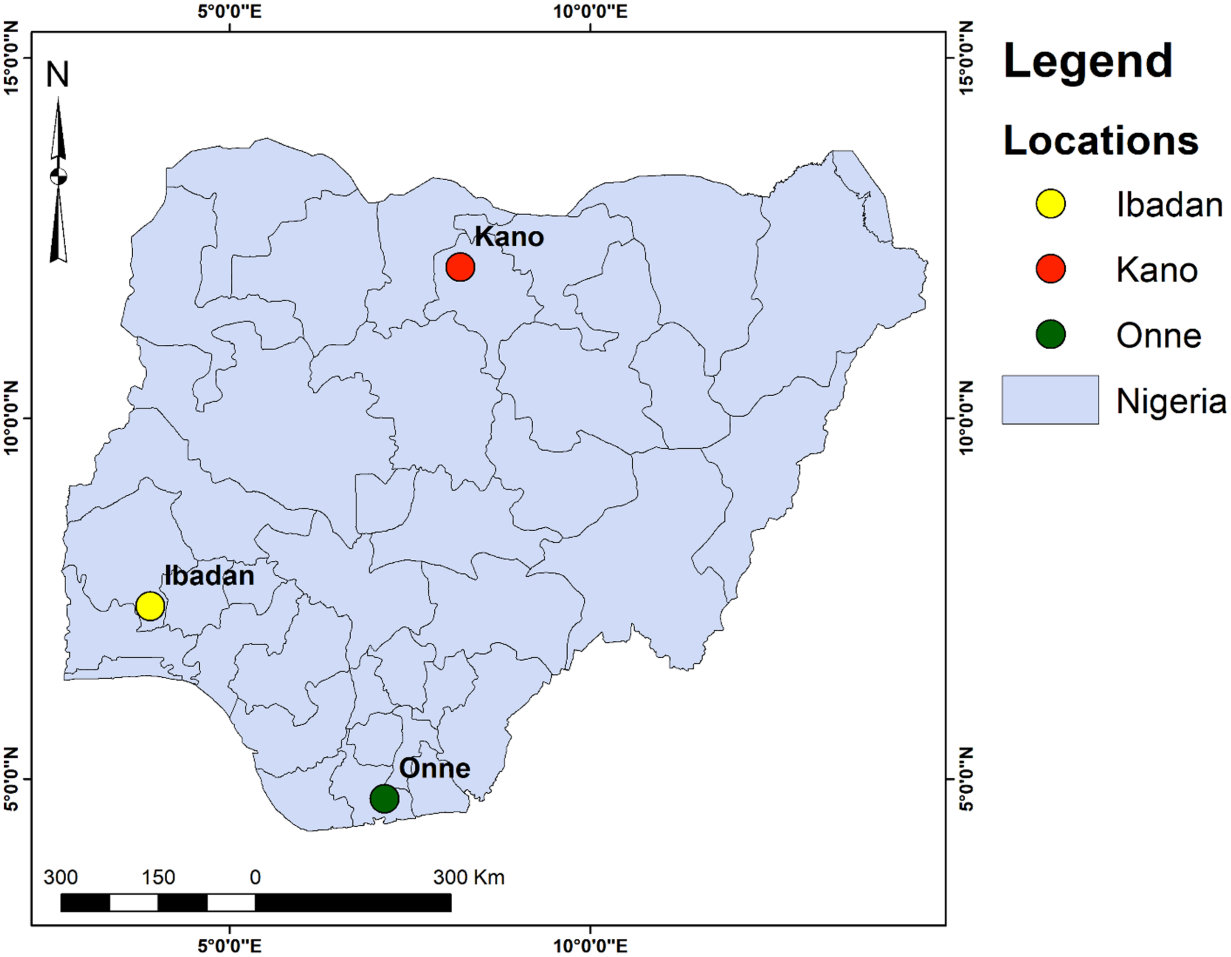
Map location of the study locations.

For this investigation, three locations were considered, representing different Nigerian states. The sites are located in Nigeria’s north (Kano), south–west (Ibadan), and south–southern (Onne) regions. The geographic locations are illustrated in Figure 1 and exhibit different climatic conditions. In Ibadan, temperatures typically range between 25 °C and 29 °C throughout the year and can occasionally rise as high as 39 °C, with an average annual rainfall of 1358 mm. The northern part of the country, including Kano, normally experiences clear skies and abundant sunshine, contributing to the area’s overall dryness. Temperatures above 29 °C are commonly recorded monthly, characterizing this location with high, scorching, and warm temperatures and minimal rainfall throughout the year. The average annual rainfall in Kano is approximately 696.4 mm. The Onne location, situated in the south–southern part of Nigeria, experiences the coolest climate with a higher amount of rainfall. The average annual temperature is approximately 26.0 °C, with average rainfall recorded at approximately 2719 mm. Rainfall can range from as low as 21.8 °C in August during the wet season to a high of 32.8 °C in January during the dry season.

### 2.2 Climatic data collection

Long-term monthly weather data, including maximum temperature (Tmax), minimum temperature (Tmin), mean relative humidity (RHM), solar radiation (Rs), and wind speed, were collected from the three locations in Nigeria (Ibadan, Kano, and Onne). The climatic data were obtained from the Nigeria Meteorological Agency (NiMET) for a 24-year period (1984–2007) at each location for the study. Missing data from any of the climatic variables considered were addressed by filling the gaps with the average of the data before and after the missing values, consistent with the approach of Halimi et al. (2023). The portion of the data that was missing was minimal (approximately 0.5%). Climatic data obtained from areas with three contrasting climatic conditions were chosen to properly evaluate the robustness (in terms of performance) of the NASA reanalysis data in accurately and precisely predicting reference evapotranspiration. The outcome would facilitate the effectiveness of integrating the NASA POWER data with ML in predicting reference evapotranspiration. The monthly weather data for all the considered climatic variables for the three locations from 1984 to 2007 were downloaded from the NASA POWER website.^1^

### 2.3 Reference evapotranspiration estimation

Reference evapotranspiration (ETo) describes the evapotranspiration from a reference surface. The reference surface is defined as a hypothetical grass reference crop with an assumed height of 0.12 m, a fixed surface resistance of 70 s m^−1^, and an albedo of 0.23 (Allen et al., 1998). This surface closely resembles an extensive area of green, well-watered grass that is actively growing and completely shading the ground. Accurate computation of ETo can be achieved by using climatic data as input. The standard method for estimating ETo is the Penman–Monteith FAO-56 method (Allen et al., 2006), which is recommended as the standard for defining and computing reference evapotranspiration. It requires data on solar radiation, air temperature (minimum and maximum), mean relative humidity, and wind speed. The equation necessary for computing reference ETo is presented in Equation 1 (Allen et al., 2006; Faloye and Alatise, 2017). The climatic data obtained from NiMET were used for ETo computation, while the climatic data from NASA POWER were used as input during predictions with the MLR and ML ETo models.


$$ET_{o}=\frac{0.408\Delta (R_{n}-G)+\gamma \frac{900}{\left(T+273\right)}u_{2}(\mathrm{es}-\mathrm{ea})}{\Delta +\gamma (1+0.34u_{2})}$$ (1)

where:

ET_o_: Reference evapotranspiration (mm d^−1^).

∆: Slope of the vapor pressure curve (kPa °C^−1^).

R_n_: Net radiation at the crop surface (MJ m^−2^ d^−1^).

G: Soil heat flux density (MJ m^−2^ d^−1^).

T: Air temperature at 2 m height (°C).

es: Saturation vapor pressure (kPa).

ea.: Actual vapor pressure (kPa).

es – ea.: Saturation vapor pressure deficit (kPa).

u_2_: Wind speed at 2 m height (m s^−1^).

*γ*: Psychrometric constant (kPa °C^−1^).

### 2.4 Multiple linear regression and machine learning algorithm development

The MLR and three different machine learning techniques, SVM, RF, and RF ensembles, were used for estimating reference evapotranspiration. The development of the SVM, RF, and RF ensemble models was implemented using MATLAB, version 2019a. The modeling approach included the use of an ML application (app) with five-fold cross-validation embedded in the MATLAB software (Paidipati et al., 2021; Faloye et al., 2023) during training. The NASA POWER climatic data served as input predictors (minimum and maximum air temperature, wind speed, and relative humidity). The Penman–Monteith (PM) ETo was computed using NiMET (ground data); minimum and maximum air temperature, solar radiation, wind speed, and relative humidity served as output (predictions). During model development, 70% of the climatic data were used for training, while the remaining 30% were used for validation. During model training, the three locations and months were included to eliminate biases in predictions during both training and validation. This approach allowed the climatic variations in the locations to be well captured by the model during training. The data from 1984 to 2000 were used for training across all months and locations, while the remaining data from 2001 to 2007 in all months and across the three locations were used for validation.

The model that generated the ETo in MATLAB during training was then exported to the workspace and applied to produce the ETo during validation. During model validation, only input (climatic data) from NASA POWER was used, while the ETo was forecasted/predicted based on this input data (NASA POWER climatic data).

#### 2.4.1 Multiple linear regression

Multiple linear regression (MLR) using the NASA POWER climatic data as input was considered for predicting the ETo. The ETo predictions were implemented in MATLAB software (version 2019a). To test the robustness of the MLR using the NASA POWER data, 70% of the dataset was used during training, while the remaining 30% was used for model validation. The developed multiple linear regression (MLR) for the ETo prediction took the form described in Equation 2 below.


ETo=β0+β1X1+β2X2+.…………+ε (2)

where ETo is the predicted reference evapotranspiration, while X_1_ and X_2_ are independent variables used for the ETo prediction.

#### 2.4.2 Support vector machine

Recently, SVM has been suggested as an innovative technique for time-series estimation, forecasting, and/or prediction, particularly in climate studies (Faloye et al., 2025). It operates based on a discriminative classifier, which is defined by a separating hyperplane. The SVM algorithm outputs an optimal hyperplane that identifies a new set of examples. In a two-dimensional space, the hyperplane divides the plane into two parts with the help of a line, thereby enabling each side to contain a different class (Bhavya et al., 2025). The SVM technique facilitates the identification of a line/hyperplane. Support vectors are data points that lie closest to the decision surface or hyperplane. The application of SVM in time-series forecasting or prediction consists of two major stages. Firstly, it uses a self-organizing feature map as the clustering algorithm to partition the entire input space into several disjoint zones. Secondly, multiple SVM kernels are tested to find the most appropriate kernel function. The choice of the SVM kernel is critical for the successful implementation of the output prediction. Several types of SVM that are commonly used include linear and quadratic (Velasco et al., 2019; Al-Mahdawi et al., 2023). Innovatively, this study tested additional SVM algorithms for ETo prediction, which include linear SVM, quadratic SVM, cubic SVM, fine Gaussian SVM, medium Gaussian SVM, and coarse Gaussian SVM. Among these SVM kernels, the best one was identified and used. From the perspective of ML, the introduction of the new kernels (cubic SVR, fine Gaussian SVR, medium Gaussian SVR, coarse Gaussian SVR), which have not been comprehensively used in previous studies for ETo prediction, demonstrates the innovation of this work. The machine learning toolbox is available in MATLAB, version 2019a. The five-fold cross-validation was used for hyperparameter value determination by locating the optimal hyperplane for SVM to avoid data overfitting. Similarly, five-fold cross-validation was used to determine the minimum leaf size for the tree. Therefore, the hyperparameters (kernel scale, box constraints, and epsilon) for the Gaussian SVM and the minimum leaf size for the DT were recorded. The kernel scale, box constraints, and epsilon were all automatically determined for the linear SVM, quadratic SVM, and cubic SVM. The kernel scale was 0.56, 2.2, and 8.9 for the fine Gaussian, medium Gaussian, and coarse Gaussian SVR, respectively.

#### 2.4.3 Decision trees

The decision tree is capable of classifying data and predicting output based on regression. The core components and ensemble of decision trees during regression are bagging and boosted-tree methods. The principle of DT ensembles is based on the combination of trees when determining output (Breiman, 1996; Tehrany et al., 2013). In this study, various decision trees (fine, medium, and coarse trees) were used, as well as their ensembles (bagged and boosted trees). The ensembles were employed in this study since they have been recognized to provide more accurate predictions (Adeodato et al., 2011). The fine, medium, coarse, bagged, and boosted decision trees were used in order to select the DT model that performs best in predicting the reference evapotranspiration from the NASA POWER data source. The optimal value of minimum leaf size used by the MATLAB software for fine, medium, coarse trees, bagged trees, and boosted trees were 4, 12, 36, 8, and 8, respectively.

### 2.5 Model statistical evaluation indices

All the data obtained for each climatic variable (maximum and minimum air temperatures, wind speed, solar radiation, and mean relative humidity), both from the ground and NASA POWER, were arranged and processed using Excel software, version 2016. In total, a dataset of 864 entries (24 years × 12 months × 3 locations) was used for the study. The performance of the ML algorithms, when integrated with the NASA POWER data, was evaluated using different model evaluation metrics based on accuracy. The metrics include root mean square error (RMSE), normalized root mean square error (NRMSE), mean absolute error (MAE), and mean square error (MSE). The equations used for the model evaluation are provided below (Equations 3–6) (Faloye et al., 2025).


$$\text{RMSE}=\sqrt{1/n\sum {\left(M_{i}-S_{i}\right)}^{2}}$$ (3)

$$\text{NRMSE}=\frac{{\sqrt{1/n\sum (M_{i}-S_{i})}}^{2}}{\bar{M}}$$ (4)

$$\mathrm{MAE}=\frac{1}{n}\sum_{i=1}^{n}|M_{i}-S_{i}|$$ (5)

$$\mathrm{MSE}=\frac{1}{n}\sum_{i=1}^{n}{|M_{i}-S_{i}|}^{2}$$ (6)

M_i_ represents the ETo obtained from ground climatic data, while S_i_ represents the ETo predicted using the MLR and ML approaches. The average value for the ETo obtained from ground-observed climatic data is depicted, while *n* is the number of meteorological observations/NASA POWER data recorded at each weather station per variable. Model evaluation metrics like RMSE, MAE, MSE, and NRMSE assist in determining the accuracy of the NASA POWER climatic data in predicting the ETo compared to the use of ground-based data. As the value of the accuracy metrics approaches zero, the output from the MLR and ML becomes more accurate. Meanwhile, as the NRMSE moves closer to 1, its accuracy increases. Based on classification, the output of a model is classified as excellent when the NRMSE is equal to or below 10% (≤0.1), “good” if it falls within 10 and 20% (≤0.2), and “fair” if it falls within 20 and 30% (0.2 ≤ NRMSE ≤0.3). It is classified as poor if the NRMSE is greater than 30% (≥0.3) (Jamieson et al., 1991). Moreover, as other accuracy terms like MSE, MAE, and RMSE approach zero, the model predictions become more accurate. The precision of the model was assessed by determining the r-squared value. As the value tends toward 1, the predictions become more perfect.

## 3 Results

### 3.1 Climatic condition of the study area

The descriptive statistics summarizing the climatic conditions of the study areas are presented in Table 1, as obtained from NiMET, which is based on ground observation. The results showed that the highest maximum temperatures were recorded in the northern part of Nigeria (Kano), followed by the southwestern part of Nigeria, while the lowest values were recorded in the south–southern part of the country (Onne). A similar pattern was observed for air temperatures (minimum and maximum) with the NASA POWER output. Moreover, the highest relative humidity (RH) was recorded at Onne, followed by Ibadan (southwestern), while the lowest values were recorded in the northern part of Nigeria (Kano), indicating that it is the driest location considered, followed by Ibadan, while Onne is the wettest. Generally, in Nigeria, temperatures rise from October until March and start declining from mid-March until September. This period defines the rainy (wet) season of the country. During the dry season, as temperatures rise, relative humidity decreases. In contrast, during the rainy season, as air temperatures decrease, relative humidity increases. The trends of relative humidity and air temperatures (minimum and maximum) obtained using NiMET data align and show a similar pattern to those obtained from the NASA POWER source (Table 2). The highest wind speed was also recorded in the northern part (Kano) based on the average value, followed by Ibadan (southwest), while the lowest average value was recorded at Onne (south–south). According to the trends and patterns obtained with air temperatures and wind speed, the highest values of solar radiation were recorded in the following increasing order: Onne < Ibadan < Kano. The solar radiation data for NASA POWER were not used directly because the platform does not yet provide the specific type of solar radiation required for the direct computation of ETo.

**Table 1:** Descriptive statistics of the ground observed weather data for the three locations.

Locations	Statistics	RH	Tmax	Tmin	WS	Tavg	Solar Radiation
KANO	MAX	81.32	41.53	26.16	7.31	33.50	26.54
	MIN	8.60	28.49	22.14	0.045	2.76	0.97
	AVERAGE	38.09	32.96	19.40	1.58	26.18	19.82
	STD	20.10	4.78	4.59	0.78	4.41	4.65
	CV	0.53	0.15	0.24	0.49	0.17	0.23
Ibadan	MAX	87.77	36.80	25.32	1.80	30.66	22.58
	MIN	47.26	26.58	16.85	0.13	23.75	8.32
	AVERAGE	74.70	31.33	22.03	0.93	26.68	15.12
	STD	8.80	2.33	1.21	0.34	1.42	2.44
	CV	0.12	0.074	0.055	0.36	0.053	0.16
ONNE	MAX	89.76	34.96	25.38	1.44	30.14	17.64
	MIN	13.84	10.20	6.25	0.25	8.23	3.99
	AVERAGE	75.94	30.14	22.37	0.92	26.26	13.03
	STD	9.70	2.41	2.10	0.19	1.99	2.34
	CV	0.13	0.080	0.094	0.20	0.076	0.18

CV is coefficient of variation, STD is standard deviation, Min is minimum value, Max is maximum value, RH is relative humidity, Tmax is maximum temperature, Tmin is minimum temperature, Tavg is average temperature, and WS is wind speed.

**Table 2:** Descriptive statistics of the NASA POWER data for the three locations.

Locations	Statistics	RH	Tmax	Tmin	WS	Tavg
Kano	MAX	82.69	43.07	33.16	1.57	23.86
	MIN	14.44	30.49	18.52	0.22	6.55
	AVERAGE	47.49	37.70	27.02	0.85	16.34
	STD	21.01	2.89	3.22	0.40	4.71
	CV	0.44	0.077	0.12	0.47	0.29
Ibadan	MAX	92.27	37.83	23.35	2.47	29.93
	MIN	56.93	28.52	12.06	0.91	22.15
	AVERAGE	84.17	31.42	19.56	1.60	25.49
	STD	7.19	1.77	2.53	0.38	1.39
	CV	0.085	0.056	0.13	0.23	0.055
Onne	MAX	92.64	34.63	25.24	1.38	28.67
	MIN	70.31	27.11	15.63	0.74	23.395
	AVERAGE	87.86	30.34	22.15	1.03	26.24
	STD	4.51	1.25	1.92	0.14	1.05
	CV	0.051	0.041	0.087	0.14	0.040

CV is coefficient of variation, STD is standard deviation, Min is minimum value, Max is maximum value, RH is relative humidity, Tmax is maximum temperature, Tmin is minimum temperature, Tavg is average temperature, and WS is wind speed.

### 3.2 Reference evapotranspiration of the study area

**Figure 2:**
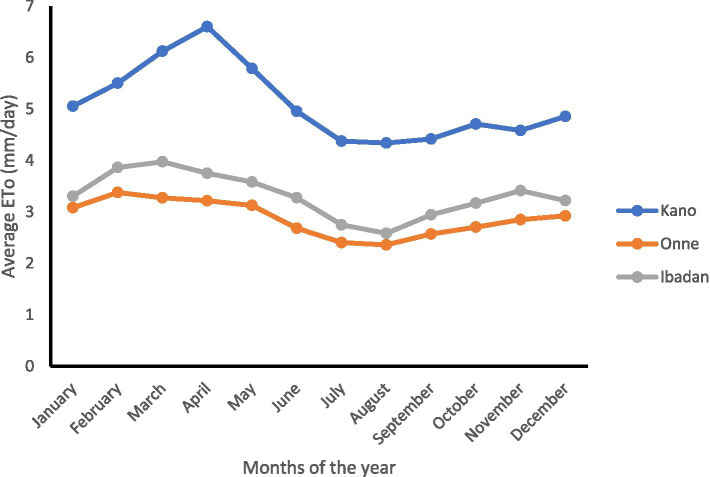
Monthly variation of the average reference evapotranspiration for Kano, Onne, and Ibadan.

Reference evapotranspiration for the three locations (Kano, Ibadan, and Onne) from 1984 to 2007 was calculated using the Penman–Monteith FAO-56 method, as shown in Figure 2. The reference evapotranspiration (ETo) had the highest values across all months (January–December), compared to those obtained for Ibadan and Onne. The ETo in Ibadan was higher than that in Onne throughout the months from 1984 to 2007. The highest ETo recorded in Kano can be attributed to the elevated air temperatures (both minimum and maximum), solar radiation, and the lowest relative humidity observed there. This observation is in agreement with the findings of Eludire et al. (2025), who posited that these variables collectively contribute to the increase in ETo values. The graphical representation (Figure 2) also showed a similar trend and pattern. There was a gradual rise in ETo from January until around April, followed by a gradual decline from around March/April until September, after which it rose again until December. The gradual decline in ETo from March/April can be attributed to the onset of the rainy season, while the increase in ETo around September can be attributed to the gradual onset of the dry season in the region.

### 3.3 Correlation between NASA POWER climatic data and ETo

The relationship between the NASA POWER climatic data and the ETo computed from the Penman–Monteith equation is shown in Table 3. The correlation table indicates that all data from NASA POWER were strongly (r mostly > 0.5) and significantly (*p* < 0.0001) correlated with ETo. Similarly, the correlation among the NASA POWER variables was very strong (*p* < 0.0001). A positive correlation was observed between the NASA POWER data and ETo for some important variables, particularly with maximum and average air temperature. In contrast, a negative correlation was recorded for relative humidity and ETo, implying that as relative humidity increases, ETo decreases, and vice versa. Overall, the significant relationship between the climatic variables and ETo showed that these parameters collectively influence ETo, justifying their inclusion in the prediction of reference evapotranspiration using multiple linear regression (MLR) and machine learning (ML) algorithms.

**Table 3:** Relationships between the NASA POWER data and reference evapotranspiration.

Parameters	RH	Tavg	Tmax	Tmin	WS
Tavg	0.015 ns				
Tmax	−0.800****	0.519****			
Tmin	0.775****	0.584****	−0.391****		
WS	0.207****	−0.169****	−0.283****	0.087*	
ETo	−0.813****	0.285****	0.860****	0.510****	0.208****

RH is relative humidity, Tmax is maximum temperature, Tmin is minimum temperature, Tavg is average temperature, and WS is wind speed; ns means non-significance, * means significance at *p*-value <0.05, **** means significance at *p* < 0.0001.

### 3.4 Model training and validation results

The result of the derived MLR equation for the model training, when data from the three locations are pooled together, is reported in Equation 7 below. The outcome revealed that the MLR predicted the ETo accurately. This is evident from the r-squared value reported in Equation 7. The value of 0.84 for the r-squared indicates that 84% of the ETo variance can be explained by the model inputs (RHM, Tmax, Tmin, and Ws). Among these parameters, the equation shows that RHM and Tmax are the most important for ETo prediction and determination. This is inferred from the strong significance (*p* < 0.0001) recorded between RHM and maximum air temperature. Moreover, the positive coefficients observed for Tmax, Tmin, and Ws confirm that as these variables increase, ETo also increases, while the negative coefficient observed for RHM indicates that as RHM decreases, ETo increases, and vice versa.


$$\begin{array}{l} \mathrm{ETo}=-1.696-0.01931\;\mathrm{RH}M^{****}+0.2037\;\mathrm{Tma}x^{****}\\ +0.0066\;\text{Tmin}+0.1096\;Ws^{*} \end{array}$$ (7)

R^2^ = 0.84.

**** mean *p* < 0.0001.

where ETo is reference evapotranspiration, RHM is relative humidity, Tmax is maximum air temperature, Tmin is minimum air temperature, and Ws is wind speed.

For the evaluation of model performance based on accuracy, the MLR performed well in predicting ETo during model training. This is evident with an NRMSE value of 0.157 (15.7%) (Table 4). When this value was compared to the results obtained using the ML, it was discovered that the ML algorithm outperformed the MLR during model training. In terms of precision, the SVM approach produced the best result during model training for both the fine Gaussian and medium Gaussian algorithms with r-squared values of 0.87. The lowest r-squared value was recorded using the coarse tree, with a value of 0.77, followed by the medium and fine trees, each producing a value of 0.82. The boosted and bagged trees (which are ensemble models) of the random forest produced improved r-squared values and accuracy terms for ETo prediction. In terms of overall model accuracy, the SVM medium Gaussian produced the best accuracy value compared to other ML algorithms. This is confirmed by the lowest MAE, MSE, RMSE, and NRMSE reported in Table 4 for the SVM medium Gaussian (MG SVM). The performance was followed by the fine Gaussian SVM, as indicated by the error metric values in Table 4.

**Table 4:** Models training results for the prediction of reference evapotranspiration.

Models	Average	MAE	MSE	RMSE	NRMSE	R-squared
Quadratic SVM	3.83	0.357	0.209	0.457	0.119	0.85
Linear SVM		0.366	0.225	0.474	0.124	0.84
Cubic SVM		0.340	0.195	0.44	0.115	0.86
FG SVM		0.362	0.258	0.438	0.114	0.87
MG SVM		0.331	0.185	0.431	0.112	0.87
Coarse SVM		0.366	0.226	0.476	0.124	0.84
Boosted tree		0.363	0.233	0.483	0.126	0.83
Bagged tree		0.360	0.223	0.473	0.123	0.84
Fine tree		0.387	0.253	0.502	0.131	0.82
Medium tree		0.384	0.256	0.506	0.132	0.82
Coarse tree		0.418	0.318	0.564	0.147	0.77
MLR		0.363	0.220	0.602	0.157	0.84

SVM is support vector machine, FG SVM is fine Gaussian support vector machine, MG SVM is medium support vector machine, MLR is multiple linear regression, MAE is mean absolute error, MSE is mean square error, RMSE is root mean square error, NRMSE is normalized root mean square error. Average is the average of daily evapotranspiration in each month across all locations from 1984 to 2000.

For model validation, the lowest precision was recorded with the medium and coarse trees, which had r-squared values of 0.6. The error metrics also showed that MAE, MSE, and NRMSE were higher when using these ML algorithms. These algorithms performed similarly to the linear and quadratic SVMs and MLR, with r-squared values of 0.69, 0.68, and 0.69, respectively. In terms of accuracy, these models produced fair results, as NRMSE values in the range of 20 to 30% are classified as “fair” in performance. However, consistent with the model training, the fine and medium SVMs yielded better results, with improved r-squared values greater than 0.7, indicating more precise predictions. Moreover, the accuracy metrics showed improved results with NRMSE values of 0.18 and 0.19, respectively (Table 5). According to classification, NRMSE values in the range of 10 to 20% are considered good. Therefore, the fine Gaussian and medium Gaussian SVMs produced good results. Similar to the accuracy results obtained from the fine and medium Gaussian algorithms, the boosted tree, which is an ensemble model of random forest, also generated more accurate results with NRMSE < 20%, compared to the ML algorithms from individual trees (fine, medium, and coarse), which had NRMSE > 20%. In addition, it is evident from Table 5 that other accuracy metrics such as MAE, MSE, and RMSE also revealed much lower values for fine Gaussian, medium Gaussian, and the boosted trees compared to the values recorded for other models (ML algorithms and MLR).

**Table 5:** Model validation results for the prediction of the reference evapotranspiration.

Models	Average	MAE	MSE	RMSE	NRMSE	R-Squared
Observed	3.37					
Linear SVM	3.67	0.504	0.567	0.753	0.22	0.69
Quad SVM	3.80	0.517	0.632	0.795	0.24	0.68
Cubic SVM	3.77	0.495	0.614	0.783	0.23	0.67
FG SVM	3.63	0.440	0.359	0.599	0.18	0.72
MG SVM	3.71	0.439	0.430	0.656	0.19	0.73
Coarse SVM	3.75	0.486	0.500	0.706	0.21	0.70
Boosted Tree	3.61	0.416	0.405	0.637	0.19	0.71
Bagged tree	3.75	0.476	0.494	0.703	0.21	0.71
MLR	3.78	0.510	0.574	0.757	0.22	0.69
Fine tree	3.76	0.502	0.553	0.744	0.22	0.70
Medium tree	3.76	0.519	0.586	0.766	0.23	0.66
Coarse tree	3.79	0.550	0.592	0.770	0.23	0.66

SVM is support vector machine, FG SVM is fine Gaussian support vector machine, MG SVM is medium support vector machine, MLR is multiple linear regression, MAE is mean absolute error, MSE is mean square error, RMSE is root mean square error, NRMSE is normalized root mean square error. Average is the average of daily evapotranspiration in each month across all locations from 2001 to 2007.

### 3.5 Evaluation of the different ML models for monthly reference evapotranspiration prediction at different locations

Tables 6–17 consist of performance metrics for different models over several months (from January to November) and for three locations (Kano, Onne, and Ibadan). The tables also show the ETo values for each month and location, which were evaluated using MAE, MSE, RMSE, and NRMSE. The ETo values provided for each month in the tables represent the actual reference evapotranspiration for that specific location during the respective month. For January in Kano, the model results show that the SVM linear model has an MAE of 0.52, MSE of 0.44, RMSE of 0.66, and NRMSE of 0.13. This means the model is off by approximately 0.52 units on average, with relatively low error values compared to other models. The ETo value for Kano in January is 5.05, which serves as a baseline for comparison with the predicted values. In January, models like “FG SVM” and “fine tree” tend to show lower MAE, MSE, RMSE, and NRMSE, indicating they perform better in predicting ETo across locations. The “coarse tree” model shows higher MAE and RMSE values in many cases, suggesting it is less accurate. The “SVM linear” model shows moderate performance, typically performing better than simpler models like “coarse tree” but not as well as others like “FG SVM.”

**Table 6:** Prediction of reference evapotranspiration for the month of January at the different locations.

Models	Kano					Onne					Ibadan				
	AVG	MAE	MSE	RMSE	NRMSE	AVG	MAE	MSE	RMSE	NRMSE	AVG	MAE	MSE	RMSE	NRMSE
ETo	5.05					3.08					3.30				
SVM linear	5.01	0.52	0.44	0.66	0.13	3.38	0.35	0.26	0.51	0.16	3.77	0.65	0.49	0.70	0.21
Quad	5.04	0.51	0.45	0.67	0.13	3.41	0.39	0.27	0.52	0.17	3.66	0.58	0.39	0.63	0.17
Cubic	5.07	0.47	0.36	0.60	0.12	3.28	0.27	0.16	0.40	0.13	3.58	0.49	0.30	0.55	0.15
FG SVM	4.93	0.33	0.24	0.49	0.097	3.26	0.20	0.11	0.33	0.11	3.58	0.43	0.31	0.55	0.15
MG SVM	5.06	0.42	0.32	0.57	0.11	3.29	0.28	0.16	0.40	0.13	3.57	0.44	0.27	0.52	0.15
Coarse SVM	5.02	0.49	0.39	0.63	0.12	3.34	0.33	0.23	0.48	0.15	3.72	0.60	0.44	0.66	0.19
Boosted	4.84	0.44	0.36	0.60	0.12	3.17	0.22	0.11	0.33	0.11	3.43	0.35	0.17	0.42	0.11
Bagged tree	5.01	0.45	0.30	0.55	0.11	3.33	0.29	0.19	0.44	0.14	3.68	0.55	0.36	0.60	0.18
Fine tree	5.04	0.46	0.34	0.58	0.11	3.22	0.22	0.12	0.34	0.11	3.49	0.38	0.18	0.42	0.12
Medium tree	5.05	0.43	0.37	0.61	0.12	3.22	0.25	0.12	0.35	0.11	3.60	0.47	0.30	0.54	0.16
Coarse tree	4.82	0.65	0.57	0.76	0.15	3.29	0.32	0.26	0.51	0.17	3.79	0.71	0.52	0.72	0.20
MLR	5.05	0.52	0.42	0.65	0.13	3.41	0.37	0.29	0.54	0.17	3.83	0.72	0.57	0.75	0.20

AVG is the average daily evapotranspiration (mm/day) in January across all locations from 1984 to 2007.

**Table 7:** Prediction of reference evapotranspiration for the month of February at the different locations.

Models	Kano					Onne					Ibadan				
	AVG	MAE	MSE	RMSE	NRMSE	AVG	MAE	MSE	RMSE	NRMSE	AVG	MAE	MSE	RMSE	NRMSE
ETo	5.32					3.44					3.85				
SVM linear	5.59	1.46	1.54	1.24	0.23	3.64	0.31	0.16	0.40	0.12	3.98	0.38	0.22	0.47	0.12
Quad	5.64	1.51	1.78	1.33	0.24	3.61	0.36	0.19	0.44	0.12	3.82	0.30	0.17	0.41	0.10
Cubic	5.51	1.51	1.80	1.34	0.24	3.56	0.29	0.13	0.36	0.10	3.81	0.32	0.20	0.44	0.12
FG SVM	5.40	1.07	0.93	0.96	0.17	3.43	0.17	0.05	0.21	0.06	3.95	0.31	0.20	0.44	0.12
MG SVM	5.55	1.44	1.56	1.25	0.23	3.49	0.28	0.11	0.33	0.10	3.81	0.31	0.17	0.41	0.10
Coarse SVM	5.50	1.42	1.40	1.18	0.21	3.61	0.32	0.16	0.40	0.11	3.95	0.36	0.22	0.47	0.12
Boosted	5.31	1.51	1.56	1.25	0.23	3.34	0.28	0.11	0.32	0.09	3.70	0.31	0.16	0.40	0.10
Bagged tree	5.36	1.36	1.31	1.15	0.22	3.57	0.26	0.09	0.31	0.09	3.87	0.33	0.16	0.40	0.11
Fine tree	5.55	1.22	1.32	1.15	0.21	3.44	0.22	0.08	0.29	0.08	3.81	0.26	0.12	0.34	0.09
Medium tree	5.55	1.57	1.93	1.39	0.25	3.43	0.28	0.11	0.34	0.10	3.93	0.32	0.15	0.39	0.10
Coarse tree	5.21	1.68	1.55	1.25	0.22	3.73	0.34	0.20	0.45	0.13	4.03	0.34	0.19	0.43	0.11
MLR	5.60	1.45	1.53	1.24	0.24	3.66	0.33	0.17	0.42	0.12	4.04	0.40	0.25	0.50	0.12

AVG is the average daily evapotranspiration (mm/day) in February across all locations from 1984 to 2007.

**Table 8:** Prediction of reference evapotranspiration for the month of March at the different locations.

Models	Kano					Onne					Ibadan				
	AVG	MAE	MSE	RMSE	NRMSE	AVG	MAE	MSE	RMSE	NRMSE	AVG	MAE	MSE	RMSE	NRMSE
ETo	5.64					3.27					3.97				
SVM linear	6.37	0.75	0.94	0.97	0.17	3.35	0.21	0.07	0.27	0.08	3.94	0.34	0.19	0.43	0.11
Quad	6.52	0.92	1.41	1.19	0.21	3.38	0.23	0.09	0.29	0.09	3.95	0.39	0.22	0.47	0.12
Cubic	6.59	0.96	1.56	1.25	0.22	3.39	0.23	0.08	0.28	0.09	3.94	0.35	0.17	0.41	0.10
FG SVM	6.09	0.62	0.51	0.72	0.13	3.33	0.20	0.07	0.26	0.08	4.07	0.23	0.09	0.30	0.08
MG SVM	6.33	0.71	0.78	0.88	0.16	3.33	0.21	0.07	0.26	0.08	3.94	0.25	0.10	0.32	0.08
Coarse SVM	6.23	0.65	0.74	0.86	0.15	3.37	0.22	0.08	0.27	0.08	3.95	0.35	0.20	0.45	0.11
Boosted	6.21	0.61	0.63	0.80	0.14	3.17	0.20	0.06	0.25	0.08	3.77	0.27	0.15	0.38	0.10
Bagged tree	6.39	0.76	0.90	0.95	0.17	3.38	0.21	0.07	0.27	0.08	3.99	0.25	0.10	0.32	0.08
Fine tree	6.44	0.83	0.96	0.98	0.17	3.25	0.17	0.05	0.21	0.06	4.01	0.19	0.06	0.25	0.06
Medium tree	6.42	0.80	0.86	0.93	0.16	3.32	0.22	0.09	0.29	0.09	3.95	0.29	0.14	0.37	0.09
Coarse tree	6.15	0.58	0.58	0.76	0.14	3.44	0.28	0.12	0.35	0.11	4.02	0.32	0.16	0.40	0.10
MLR	6.37	0.75	0.94	0.97	0.17	3.36	0.21	0.07	0.27	0.08	3.97	0.35	0.19	0.43	0.11

AVG is the average daily evapotranspiration (mm/day) in March across all locations from 1984 to 2007.

**Table 9:** Prediction of reference evapotranspiration for the month of April at the different locations.

Models	AVG	MAE	MSE	RMSE	NRMSE	AVG	MAE	MSE	RMSE	NRMSE	AVG	MAE	MSE	RMSE	NRMSE
	Kano					Onne					Ibadan				
ETo	6.60					3.22					3.75				
SVM linear	6.44	0.83	1.00	1.00	0.15	3.13	0.23	0.07	0.26	0.08	3.59	0.30	0.14	0.38	0.10
Quad	6.55	0.90	1.24	1.11	0.17	3.16	0.22	0.07	0.27	0.08	3.77	0.26	0.11	0.33	0.09
Cubic	6.78	0.85	1.27	1.13	0.17	3.18	0.21	0.07	0.27	0.08	3.81	0.24	0.11	0.33	0.09
FG SVM	6.08	0.78	1.04	1.02	0.15	3.23	0.19	0.06	0.24	0.07	3.91	0.17	0.05	0.23	0.06
MG SVM	6.41	0.80	0.89	0.94	0.14	3.20	0.20	0.07	0.26	0.08	3.82	0.24	0.09	0.30	0.08
Coarse SVM	6.27	0.84	1.03	1.01	0.15	3.18	0.19	0.06	0.24	0.08	3.64	0.28	0.13	0.36	0.10
Boosted	6.44	0.69	0.69	0.83	0.13	3.05	0.24	0.08	0.28	0.09	3.59	0.23	0.10	0.32	0.09
Bagged tree	6.56	0.72	0.68	0.83	0.13	3.21	0.16	0.04	0.21	0.07	3.73	0.22	0.09	0.30	0.08
Fine tree	6.78	0.53	0.53	0.73	0.11	3.22	0.16	0.05	0.22	0.07	3.77	0.20	0.07	0.26	0.07
Medium tree	6.61	0.71	0.62	0.79	0.12	3.14	0.19	0.05	0.23	0.07	3.81	0.24	0.09	0.30	0.08
Coarse tree	6.15	0.81	1.02	1.01	0.15	3.19	0.19	0.06	0.25	0.08	3.73	0.25	0.11	0.33	0.09
MLR	6.43	0.83	0.99	0.99	0.15	3.13	0.21	0.06	0.25	0.08	3.61	0.29	0.14	0.37	0.10

AVG is the average daily evapotranspiration (mm/day) in April across all locations from 1984 to 2007.

**Table 10:** Prediction of reference evapotranspiration for the month of May at different locations.

Models	Kano					Onne					Ibadan				
	AVG	MAE	MSE	RMSE	NRMSE	AVG	MAE	MSE	RMSE	NRMSE	AVG	MAE	MSE	RMSE	NRMSE
ETo	5.79					3.12					3.58				
SVM linear	6.03	0.67	1.02	1.01	0.17	2.97	0.24	0.09	0.30	0.10	3.26	0.37	0.20	0.44	0.12
Quad	6.10	0.71	1.24	1.11	0.19	2.97	0.25	0.10	0.32	0.10	3.37	0.31	0.15	0.38	0.11
Cubic	6.05	0.70	1.12	1.06	0.18	2.97	0.26	0.11	0.33	0.10	3.48	0.26	0.11	0.32	0.09
FG SVM	5.77	0.52	0.63	0.79	0.14	3.09	0.22	0.07	0.26	0.08	3.65	0.27	0.11	0.33	0.09
MG SVM	5.99	0.63	0.87	0.93	0.16	3.03	0.25	0.09	0.30	0.10	3.53	0.26	0.10	0.32	0.09
Coarse SVM	5.89	0.67	0.89	0.95	0.16	3.02	0.23	0.08	0.28	0.09	3.27	0.37	0.19	0.44	0.12
Boosted	5.89	0.56	0.77	0.88	0.15	2.91	0.26	0.11	0.33	0.11	3.37	0.30	0.13	0.35	0.10
Bagged tree	6.03	0.65	0.96	0.98	0.17	3.07	0.20	0.06	0.24	0.08	3.49	0.24	0.09	0.30	0.08
Fine tree	6.13	0.52	0.95	0.98	0.17	3.11	0.18	0.05	0.23	0.07	3.68	0.19	0.07	0.26	0.07
Medium tree	5.96	0.72	0.95	0.97	0.17	3.04	0.20	0.06	0.25	0.08	3.53	0.28	0.11	0.33	0.09
Coarse tree	6.15	0.73	0.97	0.98	0.17	2.97	0.28	0.11	0.33	0.11	3.44	0.26	0.11	0.33	0.09
MLR	6.02	0.66	1.00	1.00	0.17	2.97	0.24	0.09	0.30	0.10	3.28	0.36	0.18	0.43	0.12

AVG is the average daily evapotranspiration (mm/day) in May across all locations from 1984 to 2007.

**Table 11:** Prediction of reference evapotranspiration for the month of June at different locations.

Models	AVG	MAE	MSE	RMSE	NRMSE	AVG	MAE	MSE	RMSE	NRMSE	AVG	MAE	MSE	RMSE	NRMSE
	Kano					Onne					Ibadan				
ETo	4.96					2.68					3.27				
SVM linear	5.47	0.60	0.48	0.69	0.14	2.83	0.33	0.18	0.43	0.16	3.03	0.29	0.13	0.36	0.11
Quad	5.28	0.48	0.32	0.57	0.11	2.81	0.34	0.20	0.45	0.17	3.10	0.31	0.17	0.41	0.13
Cubic	5.09	0.40	0.21	0.45	0.09	2.75	0.32	0.18	0.43	0.16	3.26	0.28	0.13	0.36	0.11
FG SVM	4.96	0.22	0.09	0.30	0.06	2.81	0.28	0.15	0.39	0.15	3.27	0.29	0.13	0.36	0.11
MG SVM	5.12	0.43	0.24	0.49	0.10	2.78	0.31	0.17	0.41	0.15	3.24	0.30	0.14	0.38	0.12
Coarse SVM	5.38	0.53	0.38	0.62	0.13	2.86	0.33	0.19	0.44	0.16	3.05	0.31	0.18	0.42	0.13
Boosted	4.83	0.35	0.20	0.44	0.09	2.70	0.31	0.17	0.41	0.15	3.06	0.30	0.16	0.40	0.12
Bagged tree	5.15	0.39	0.24	0.49	0.10	2.83	0.29	0.16	0.39	0.15	3.21	0.25	0.09	0.31	0.09
Fine tree	5.09	0.37	0.22	0.47	0.10	2.84	0.26	0.12	0.34	0.13	3.31	0.36	0.19	0.44	0.13
Medium tree	5.26	0.49	0.46	0.68	0.14	2.90	0.36	0.20	0.44	0.16	3.19	0.32	0.16	0.40	0.12
Coarse tree	5.76	1.00	1.23	1.11	0.22	2.81	0.35	0.19	0.44	0.16	3.20	0.29	0.14	0.37	0.11
MLR	5.48	0.60	0.49	0.70	0.14	2.84	0.33	0.18	0.43	0.16	3.06	0.31	0.17	0.42	0.13

AVG is the average daily evapotranspiration (mm/day) in June across all locations from 1984 to 2007.

**Table 12:** Prediction of reference evapotranspiration for the month of July at different locations.

Models	Kano					Onne					Ibadan				
	AVG	MAE	MSE	RMSE	NRMSE	AVG	MAE	MSE	RMSE	NRMSE	AVG	MAE	MSE	RMSE	NRMSE
ETo	4.38					2.40					2.75				
SVM linear	4.73	0.45	0.48	0.69	0.16	2.70	0.36	0.19	0.43	0.18	2.88	0.33	0.16	0.40	0.14
Quad	4.52	0.36	0.29	0.53	0.11	2.63	0.32	0.16	0.40	0.16	2.90	0.37	0.20	0.44	0.16
Cubic	4.15	0.37	0.18	0.42	0.09	2.56	0.31	0.14	0.37	0.16	2.98	0.35	0.19	0.44	0.16
FG SVM	4.40	0.15	0.03	0.17	0.04	2.49	0.22	0.08	0.28	0.12	2.95	0.31	0.17	0.41	0.15
MG SVM	4.34	0.27	0.11	0.34	0.08	2.60	0.30	0.13	0.37	0.15	2.87	0.31	0.16	0.40	0.15
Coarse SVM	4.66	0.41	0.37	0.61	0.14	2.72	0.38	0.20	0.45	0.19	2.94	0.35	0.18	0.42	0.15
Boosted	4.36	0.33	0.21	0.46	0.10	2.47	0.27	0.10	0.31	0.13	2.75	0.31	0.15	0.39	0.14
Bagged tree	4.66	0.39	0.44	0.67	0.15	2.60	0.29	0.15	0.39	0.16	2.88	0.30	0.15	0.39	0.14
Fine tree	4.42	0.33	0.26	0.51	0.11	2.50	0.20	0.08	0.28	0.12	2.89	0.28	0.16	0.40	0.14
Medium tree	4.55	0.47	0.49	0.70	0.16	2.51	0.22	0.09	0.30	0.12	2.89	0.31	0.17	0.42	0.15
Coarse tree	4.71	0.41	0.57	0.76	0.17	2.60	0.32	0.15	0.39	0.16	2.84	0.39	0.22	0.47	0.17
MLR	4.75	0.46	0.48	0.70	0.15	2.70	0.36	0.19	0.44	0.18	2.91	0.33	0.16	0.40	0.15

AVG is the average daily evapotranspiration (mm/day) in July across all locations from 1984 to 2007.

**Table 13:** Prediction of reference evapotranspiration for the month of August at different locations.

Models	AVG	MAE	MSE	RMSE	NRMSE	AVG	MAE	MSE	RMSE	NRMSE	AVG	MAE	MSE	RMSE	NRMSE
	Kano					Onne					Ibadan				
ETo	4.34					2.36					2.58				
SVM linear	4.16	0.41	0.27	0.52	0.12	2.73	0.42	0.30	0.55	0.23	2.89	0.44	0.26	0.51	0.20
Quad	4.26	0.25	0.11	0.33	0.08	2.67	0.38	0.27	0.52	0.22	2.89	0.43	0.25	0.50	0.19
Cubic	4.39	0.18	0.04	0.21	0.05	2.63	0.37	0.26	0.51	0.21	2.90	0.40	0.24	0.49	0.19
FG SVM	4.37	0.16	0.04	0.19	0.04	2.46	0.26	0.16	0.40	0.17	2.79	0.28	0.15	0.38	0.15
MG SVM	4.35	0.20	0.05	0.23	0.05	2.63	0.36	0.24	0.49	0.21	2.81	0.34	0.18	0.42	0.16
Coarse SVM	4.16	0.39	0.24	0.49	0.11	2.74	0.43	0.31	0.56	0.24	2.95	0.49	0.31	0.56	0.22
Boosted	4.25	0.21	0.06	0.25	0.06	2.51	0.27	0.18	0.43	0.18	2.76	0.33	0.17	0.42	0.16
Bagged tree	4.38	0.21	0.06	0.24	0.06	2.60	0.30	0.21	0.46	0.20	2.89	0.40	0.26	0.51	0.20
Fine tree	4.46	0.19	0.06	0.25	0.06	2.47	0.22	0.14	0.38	0.16	2.85	0.31	0.19	0.44	0.17
Medium tree	4.29	0.33	0.19	0.44	0.10	2.51	0.24	0.16	0.40	0.17	2.93	0.43	0.35	0.59	0.23
Coarse tree	4.28	0.30	0.15	0.39	0.09	2.63	0.31	0.24	0.49	0.21	2.88	0.41	0.30	0.54	0.21
MLR	4.19	0.39	0.25	0.50	0.12	2.74	0.42	0.30	0.55	0.23	2.92	0.46	0.29	0.54	0.21

AVG is the average daily evapotranspiration (mm/day) in August across all locations from 1984 to 2007.

**Table 14:** Prediction of reference evapotranspiration for the month of September at different locations.

Models	Kano					Onne					Ibadan				
	AVG	MAE	MSE	RMSE	NRMSE	AVG	MAE	MSE	RMSE	NRMSE	AVG	MAE	MSE	RMSE	NRMSE
ETo	4.42					2.57					2.94				
SVM linear	4.44	0.56	0.65	0.81	0.18	2.76	0.22	0.08	0.27	0.11	3.00	0.33	0.16	0.40	0.14
Quad	4.53	0.44	0.45	0.67	0.15	2.75	0.21	0.07	0.26	0.10	3.04	0.35	0.17	0.42	0.14
Cubic	4.66	0.41	0.41	0.64	0.15	2.66	0.19	0.05	0.22	0.09	3.20	0.38	0.22	0.47	0.16
FG SVM	4.45	0.30	0.31	0.55	0.13	2.64	0.16	0.04	0.20	0.08	3.20	0.34	0.18	0.43	0.14
MG SVM	4.45	0.36	0.35	0.59	0.13	2.67	0.18	0.05	0.22	0.09	3.16	0.39	0.22	0.47	0.16
Coarse SVM	4.43	0.53	0.59	0.77	0.17	2.79	0.25	0.09	0.30	0.12	3.00	0.33	0.16	0.40	0.13
Boosted	4.36	0.40	0.36	0.60	0.14	2.58	0.18	0.04	0.21	0.08	3.04	0.33	0.15	0.39	0.13
Bagged tree	4.55	0.39	0.38	0.61	0.14	2.69	0.17	0.05	0.22	0.08	3.09	0.30	0.14	0.37	0.13
Fine tree	4.60	0.42	0.50	0.70	0.16	2.63	0.21	0.07	0.26	0.10	3.19	0.35	0.24	0.49	0.17
Medium tree	4.64	0.52	0.58	0.76	0.17	2.61	0.25	0.09	0.30	0.12	3.23	0.39	0.23	0.48	0.16
Coarse tree	4.58	0.59	0.86	0.92	0.21	2.74	0.22	0.08	0.28	0.11	3.22	0.42	0.24	0.49	0.17
MLR	4.48	0.55	0.64	0.80	0.18	2.77	0.23	0.08	0.28	0.11	3.03	0.33	0.16	0.40	0.14

AVG is the average daily evapotranspiration (mm/day) in September across all locations from 1984 to 2007.

**Table 15:** Prediction of reference evapotranspiration for the month of October at different locations.

Models	AVG	MAE	MSE	RMSE	NRMSE	AVG	MAE	MSE	RMSE	NRMSE	AVG	MAE	MSE	RMSE	NRMSE
	Kano					Onne					Ibadan				
ETo	4.71					2.70					3.17				
SVM linear	4.70	0.62	0.68	0.82	0.17	2.85	0.19	0.08	0.28	0.10	3.06	0.28	0.11	0.34	0.11
Quad	4.71	0.49	0.40	0.63	0.13	2.88	0.22	0.10	0.32	0.12	3.14	0.28	0.11	0.33	0.10
Cubic	4.77	0.44	0.38	0.62	0.13	2.84	0.21	0.09	0.30	0.11	3.14	0.28	0.11	0.33	0.10
FG SVM	4.76	0.30	0.16	0.40	0.09	2.91	0.24	0.09	0.31	0.11	3.24	0.24	0.08	0.29	0.09
MG SVM	4.65	0.53	0.44	0.67	0.14	2.85	0.21	0.08	0.29	0.11	3.08	0.28	0.12	0.35	0.11
Coarse SVM	4.69	0.59	0.61	0.78	0.17	2.89	0.22	0.09	0.30	0.11	3.05	0.28	0.12	0.34	0.11
Boosted	4.58	0.57	0.58	0.76	0.16	2.77	0.20	0.07	0.26	0.10	3.04	0.29	0.12	0.35	0.11
Bagged tree	4.76	0.45	0.34	0.59	0.12	2.86	0.21	0.08	0.29	0.11	3.26	0.24	0.09	0.30	0.09
Fine tree	4.85	0.42	0.35	0.59	0.13	2.83	0.20	0.07	0.27	0.10	3.34	0.28	0.13	0.36	0.11
Medium tree	4.90	0.71	1.03	1.01	0.22	2.91	0.26	0.11	0.34	0.12	3.29	0.23	0.08	0.28	0.09
Coarse tree	4.67	0.63	0.76	0.87	0.18	2.87	0.20	0.09	0.29	0.11	3.32	0.31	0.14	0.37	0.12
MLR	4.76	0.61	0.65	0.81	0.17	2.85	0.19	0.08	0.28	0.10	3.09	0.27	0.11	0.33	0.10

AVG is the average daily evapotranspiration (mm/day) in October across all locations from 1984 to 2007.

**Table 16:** Prediction of reference evapotranspiration for the month of November at different locations.

Models	Kano					Onne					Ibadan				
	AVG	MAE	MSE	RMSE	NRMSE	AVG	MAE	MSE	RMSE	NRMSE	AVG	MAE	MSE	RMSE	NRMSE
ETo	4.58					2.85					3.41				
SVM linear	4.78	0.52	0.38	0.62	0.14	3.01	0.23	0.09	0.29	0.10	3.25	0.27	0.13	0.36	0.11
Quad	4.58	0.38	0.20	0.45	0.10	3.05	0.27	0.11	0.34	0.12	3.34	0.27	0.12	0.35	0.10
Cubic	4.66	0.36	0.17	0.42	0.09	3.09	0.29	0.13	0.35	0.12	3.19	0.31	0.17	0.41	0.12
FG SVM	4.50	0.25	0.09	0.30	0.07	3.02	0.23	0.08	0.29	0.10	3.35	0.28	0.12	0.35	0.10
MG SVM	4.61	0.35	0.17	0.41	0.09	3.08	0.28	0.12	0.34	0.12	3.20	0.30	0.16	0.40	0.12
Coarse SVM	4.79	0.48	0.33	0.57	0.12	3.04	0.26	0.10	0.32	0.11	3.23	0.29	0.14	0.38	0.11
Boosted	4.43	0.28	0.12	0.35	0.08	2.96	0.20	0.06	0.25	0.09	3.17	0.32	0.18	0.42	0.12
Bagged tree	4.86	0.42	0.23	0.48	0.11	3.07	0.25	0.09	0.31	0.11	3.37	0.27	0.11	0.34	0.10
Fine tree	4.77	0.29	0.17	0.41	0.09	3.02	0.21	0.08	0.29	0.10	3.55	0.28	0.14	0.37	0.11
Medium tree	4.69	0.39	0.19	0.44	0.10	3.06	0.27	0.11	0.34	0.12	3.44	0.30	0.13	0.35	0.10
Coarse tree	4.70	0.51	0.47	0.69	0.15	3.10	0.28	0.12	0.35	0.12	3.39	0.37	0.19	0.43	0.13
MLR	4.81	0.52	0.38	0.62	0.13	3.02	0.24	0.09	0.30	0.10	3.30	0.26	0.12	0.35	0.10

AVG is the average daily evapotranspiration (mm/day) in November across all locations from 1984 to 2007.

**Table 17:** Prediction of reference evapotranspiration for the month of December at different locations.

Models	AVG	MAE	MSE	RMSE	NRMSE	AVG	MAE	MSE	RMSE	NRMSE	AVG	MAE	MSE	RMSE	NRMSE
	Kano					Onne					Ibadan				
ETo	4.85					2.92					3.22				
SVM linear	4.69	0.55	0.41	0.64	0.13	3.18	0.30	0.20	0.45	0.15	3.43	0.32	0.13	0.37	0.11
Quad	4.64	0.54	0.34	0.58	0.12	3.22	0.37	0.25	0.50	0.17	3.47	0.38	0.19	0.43	0.13
Cubic	4.72	0.46	0.25	0.50	0.10	3.19	0.31	0.20	0.44	0.15	3.33	0.24	0.09	0.29	0.09
FG SVM	4.67	0.23	0.08	0.29	0.06	3.07	0.22	0.12	0.34	0.12	3.36	0.19	0.07	0.26	0.08
MG SVM	4.77	0.42	0.21	0.46	0.09	3.21	0.31	0.20	0.45	0.15	3.34	0.25	0.09	0.31	0.09
Coarse SVM	4.74	0.50	0.34	0.58	0.12	3.16	0.30	0.19	0.44	0.15	3.40	0.31	0.13	0.36	0.11
Boosted	4.64	0.40	0.18	0.43	0.09	3.08	0.25	0.15	0.38	0.13	3.22	0.19	0.07	0.26	0.08
Bagged tree	4.88	0.35	0.19	0.43	0.09	3.17	0.28	0.20	0.44	0.15	3.39	0.25	0.10	0.31	0.10
Fine tree	4.75	0.29	0.12	0.34	0.07	3.05	0.21	0.14	0.38	0.13	3.30	0.17	0.06	0.25	0.08
Medium tree	4.73	0.46	0.25	0.50	0.10	3.16	0.29	0.15	0.39	0.13	3.35	0.23	0.09	0.29	0.09
Coarse tree	4.68	0.56	0.63	0.80	0.16	3.18	0.29	0.16	0.41	0.14	3.58	0.43	0.24	0.49	0.15
MLR	4.73	0.53	0.39	0.62	0.13	3.21	0.32	0.22	0.46	0.16	3.49	0.36	0.17	0.41	0.13

AVG is the average daily evapotranspiration (mm/day) in December across all locations from 1984 to 2007.

For January, the results of the analysis show that the SVM linear model performs relatively well in predicting ETo values, with consistently low MAE and RMSE values for each location. The results for Quad are similar to those of SVM linear, with minor differences in the MAE and RMSE values. For example, in Kano, the MAE is 0.51, slightly worse than the SVM linear model’s 0.52, but the results are still reasonable. The Cubic model shows a slight improvement in terms of RMSE (lower than some other models) in Onne and Ibadan. The FG SVM performed excellently with very low MAE and RMSE values, especially in Kano (MAE = 0.33, RMSE = 0.49). Similar to FG SVM, MG SVM performs reasonably well across all locations, with low RMSE and MAE values, making it another reliable choice for ETo prediction. Overall, in January, the best performing model was the FG SVM, which stood out with the lowest error metrics (MAE, MSE, RMSE, NRMSE), particularly in Kano, followed by MG SVM. The worst performing models in January were the coarse tree and boosted models, which showed higher errors, particularly in the RMSE metric.

In February, the SVM linear model performs somewhat well, though the MAE and RMSE are a bit higher than in January. FG SVM continues to perform strongly, especially in Onne and Ibadan. The coarse tree again shows poor performance in terms of MAE and RMSE, especially in Kano and Ibadan. The best performing model was identified as FG SVM, showing the best results overall, with consistently low MAE, MSE, and RMSE across all locations. Again, the worst performing models were the coarse tree and boosted models for February, which continued to exhibit higher MAE and RMSE.

In the month of March, the SVM linear model produced the following results: MAE = 0.75, MSE = 0.94, RMSE = 0.97 (Kano). A noticeable increase in error compared to previous months was observed. The FG SVM performs well across all locations, although the error increases slightly compared to earlier months, while the coarse tree shows high error values, continuing to be a less accurate model. Overall, this month, the FG SVM remains the best performing model, although its MAE and RMSE are slightly higher than in previous months, while the worst performing models were SVM linear and coarse tree.

In the month of April, the SVM linear produced the following statistics: MAE = 0.83, MSE = 1.00, RMSE = 1.00 (Kano). The performance decreased significantly in comparison to earlier months. This month, the FG SVM and MG SVM were the models that performed best across all locations with consistent MAE and RMSE values. Overall, the best performing model this month was FG SVM, which continues to show the best performance, while the worst performing models were SVM linear and coarse tree.

However, in the month of May, the SVM linear showed better performance than in April but still had higher MAE and RMSE compared to FG SVM. FG SVM and MG SVM remained the best models, with low error metrics, while the coarse tree showed poor results in terms of MAE and RMSE. Overall, the models that performed best in the month of May across the three locations were FG SVM and MG SVM, while the worst performing models were coarse tree and SVM linear.

From June to December, similar trends continued; the ETo values fluctuated across months for each location. The FG SVM remained one of the best performers in terms of low MAE, MSE, RMSE, and NRMSE. The coarse tree continued to show higher error metrics, indicating poorer performance. The boosted tree and bagged tree models also showed moderate performance, with higher error values than FG SVM. Additionally, the SVM linear performed decently but tended to lag behind FG SVM and MG SVM in terms of prediction accuracy.

**Figure 3:**
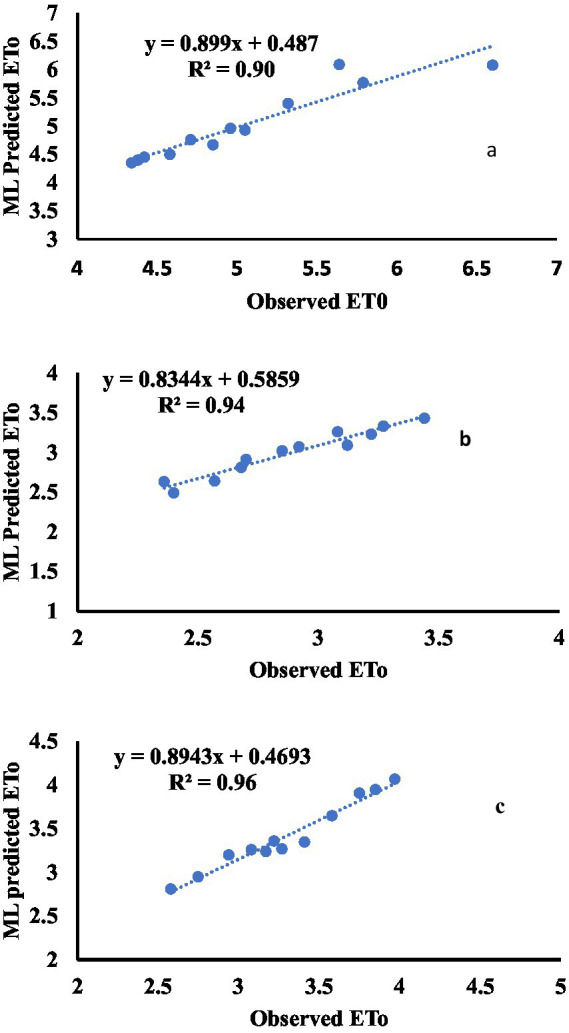
Relationships between observed ETo and the ML predicted ETo using FG SVM for Kano **(a)**, Ibadan **(b)**, and Onne **(c)** locations.

The graphical illustration of the relationship between the observed ETo and the ML prediction using FG SVM (the best algorithm identified) is shown in Figures 3a–c. The r-squared values, which represent the precision evaluation of the models, revealed that the model was excellent in predicting ETo, with r-squared values ranging from 0.90 to 0.96. The magnitude of the prediction increased from the northern part (Kano) to the southwestern (Ibadan) and south–southern (Onne regions). The model’s precision (FG SVM) at Ibadan and Onne was similar.

## 4 Discussion

In the present study, several machine learning algorithms were tested and evaluated to identify the most suitable ML model for estimating ETo values in areas with contrasting weather conditions in Nigeria. Accurate estimation of ETo is fundamental for calculating crop evapotranspiration (ETc). This can be challenging in areas where weather data are scarce. Thus, integrating ML and remotely sensed data could offer a solution. Accordingly, the SVM FG model exhibited the highest accuracy, while the coarse tree showed the weakest prediction success. It was observed that Tmax, Tmin, and RHmean input variables had the most significant effect on ETo estimation. This was confirmed by the strong and significant relationships between these variables and ETo values, which agrees with the findings of Pal and Deswal (2009), who employed ML models (decision trees) to predict ETo in California using additional input variables (Tmax, Tmin, RHmean, and solar radiation). In the present study, Tmax, RHmean, and Tmin were the major indicators of ETo. Lower relative humidity and higher temperatures were recorded in the northern part of Nigeria, while the opposite was true in the southern part (Adeboye et al., 2009). The findings were supported by Kim et al. (2006), who noted that an increase in air moisture content causes relative humidity to have a greater impact in wetter locations. They further suggested that as the aridity index increases, air moisture content becomes constrained, reducing its effects. Temperature and relative humidity were identified as the most important predictors of ETo in a study conducted by Aschale et al. (2023). Eslamian et al. (2011) investigated the effect of weather parameters on ETo estimation in Esfahan province, concluding that Tmin, Sh, and RHmean were effective parameters for ETo estimation in this region.

The FG SVM and the fine tree model successfully predicted the ETo values, while the bagged tree showed moderate performance (Raza et al., 2023). The models used in this study accurately predicted ETo despite the limited weather variables employed, compared to the broader range of variables including solar radiation used by Pal and Deswal (2009). The strong predictions observed in this study can be attributed to the careful selection of the ML algorithm, which facilitated the identification of the best model among those tested. The range of RMSE was lower than the 0.41 to 0.76 mm reported by Wang et al. (2022) using the random forest algorithm, iterative dichotomiser, and gradient boosting algorithm. Similarly, the RMSE values in our study were lower than the 0.28 mm reported by Mehdizadeh et al. (2021) using the Adaptive Neuro Fuzzy Inference System. Moreover, Farag et al. (2025) recently investigated various approaches for predicting ETo using different ML algorithms and found RMSE values of 0.52 mm for random forest, which outperformed other models like K-nearest neighbors (KNN) and decision tree (DT). Notably, the different SVM and decision tree algorithms used in this study revealed that the FG SVM outperformed the other models, which had not been tested in previous studies, highlighting the innovation of this study. The RMSE, MSE, and MAE values obtained using the FG SVM indicated that this model is reliable in terms of accuracy when compared to the RMSE values from related studies, as detailed above. In most cases, the FG SVM and MG SVM performed similarly in predicting ETo. While the medium SVM strikes a fair balance between performance and complexity, the fine SVM allows the model to learn from a greater number of features. This combination of attributes in both FG and MG results in close, accurate predictions by enhancing the model’s ability to predict the output with new inputs effectively. The outcomes of this study suggest that both FG SVM and MG can be used interchangeably for ETo prediction.

The capacity of this model was tested in areas with contrasting weather conditions in Nigeria, confirming the robustness of this result. Another innovation of this study is the integration of remotely sensed data (NASA POWER) with machine learning, which is sufficient for the accurate prediction of ETo in regions where weather data are missing or lacking. This aspect, in particular, highlights the innovation of the study, as noted in previous studies (Raza et al., 2023; Kartal, 2024; Kim et al., 2022). Moreover, in terms of precision, the FG model also performed well, with a range of values from 0.94 to 0.96 reported in this study, similar to the range of 0.87 to 0.99 reported by Bhavya et al. (2025) and close to a value of 0.98 reported by Raza et al. (2023). The degree of detail in a decision tree’s structure can affect its accuracy. Fine Gaussian (FG) decision trees can produce more accurate predictions because they are more comprehensive and divide the data into smaller subsets (Blockeel et al., 2023). This is due to their ability to identify more nuanced relationships and patterns in the data, leading to more accurate classifications. Coarse decision trees, on the other hand, may oversimplify the data and yield less accurate predictions (Blockeel et al., 2023). To achieve high accuracy, decision trees must strike a balance between simplicity and complexity. Considering the overall performance of the model, the accuracy indicates that the performance of the ML model depends on the month of the year and location, which can be attributed to the values of the meteorological variables, such as relative humidity, temperature, and wind speed, input into the model (Aly et al., 2024). The accurate and precise results obtained in this study can be attributed to the choice of ML algorithms selected and the training of the models using NASA POWER data. Therefore, in developing ML models with NASA POWER data, the climatic data must be trained and validated before use.

## 5 Conclusion

This study evaluated the performance of various support vector machine and decision tree (DT)-based machine learning models for the prediction of ETo in Nigeria. The study also explained the relationship between the input parameters from NASA POWER and the ETo values computed using ground-based weather variables, yielding favorable results, thus confirming the reliability of the input source (NASA POWER). Generally, satisfactory results were obtained mostly using the ML models compared to the output from the multiple linear regression (MLR) approach. Among the ML models tested, the FG and MG SVM provided the best results during model training and validation, while the coarse tree prediction yielded the worst outcomes. When evaluating the model’s performance across locations, it was discovered that the FG SVM consistently retained its best predictions. Therefore, the FG SVM is recommended for the prediction of ETo across different regions of Nigeria (north, southwest, and south–south), even when climatic data from ground sources are unavailable. Accurate estimation of ETo will be possible by integrating ML (FG SVM) with NASA POWER data, which has been confirmed to be accurate and precise after the model evaluation in our study.

It is therefore recommended to ensure that the remotely sensed data (NASA) are properly trained using ML techniques. Poorly trained data can diminish the accuracy and precision of ETo estimations, which can lead to misjudgments in estimating water use by crops. This study integrated NASA POWER data with different ML models and evaluated their results; therefore, further research is needed using NASA POWER and other remote sensing data sources integrated with ML in areas with similar or varying weather conditions.

## Data Availability

The raw data supporting the conclusions of this article will be made available by the authors, without undue reservation.

## Footnotes

1
https://power.larc.nasa.gov/
